# Efficiency of sequencing batch reactor for removal of organic matter in the effluent of petroleum wastewater

**DOI:** 10.1016/j.dib.2018.06.094

**Published:** 2018-07-04

**Authors:** Abdolkazem Neisi, Shirin Afshin, Yousef Rashtbari, Ali Akbar Babaei, Yusef Omidi Khaniabadi, Anvar Asadi, Mohammad Shirmardi, Mehdi Vosoughi

**Affiliations:** a Environmental Technologies Research Center, Ahvaz Jundishapur University of Medical Sciences, Ahvaz, Iran; b Department of Environmental Health Engineering, School of Public Health, Student research committee, Ardabil University of Medical Sciences, Ardabil, Iran; c Department of Environmental Health Engineering, School of Public Health, Environmental Technologies Research Center, Ahvaz Jundishapur University of Medical Sciences, Ahvaz, Iran; d Department of Environmental Health, Health Care System of Karoon, Ahvaz Jundishapur University of Medical Sciences, Ahvaz, Iran; e Research Center for Environmental Determinants of Health (RCEDH), Kermanshah University of Medical Sciences, Kermanshah, Iran; f Environmental Health Research Center, Health Research Institute, Babol University of Medical Sciences, Babol, Iran; g Social Determinants of Health Research Center, Ardabil University of Medical Sciences, Ardabil, Iran

**Keywords:** Methyl Tert-Butyl Ether (MTBE), Sequencing batch reactor (SBR), Chemical oxygen demand (COD), Biodegradation

## Abstract

The main aim of this research was to study the biodegradation of Methyl Tertiary Butyl Ether (MTBE) using aerobic sequencing batch reactor (SBR) at a pilot-Scale. The reactor was made of a 3 mm-thick glass cylinder with an internal diameter of 12 cm and height of 60 cm. SBR operated in five phases. The first phase was filling the reactor for about 10 min. the second phase was the main reactor for biological treatment of petroleum wastewater about 21.55 h. The third phase was the sedimentation (1 h). The fourth phase was decanting from the reactor for about 10 min. The last phase consisted of idle for about 45 min. The experiments showed that the mixed microbial mass is able to degrade high concentration of methanol up to 250 mg/l, and concentration of MTBE up to 70 mg/l for a 24 h cycle. However, the mixed microbial mass is not able to degrade MTBE with concentration more than 70 mg/l. Microorganisms were generally isolated from Fajr petrochemical wastewater treatment plant. Analysis showed that the mixed microbial mass able to biodegradation of COD up to 1350 mg/l in effluent. Aerobic SBR can be used for biological treatment of the petroleum wastewater containing pollutants such as methanol, MTBE with a promising efficiency.

**Specifications Table**

**Table:** 

Subject area	Environmental Engineering
More specific subject area	– Industrial effluent treatment – Wastewater technology
Type of data	Tables, Figures and Text file
How data was acquired	– Aerobic sequencing batch reactor (SBR) in a pilot-Scale was used for collect data of biodegradation efficiency. – Microorganisms for biodegradation were isolated from the real petrochemical wastewater treatment plant. – All physical and chemical experiments were performed based on the standard methodology for water and wastewater experiments. – Gas Chromatograph (Agilent model), double beam spectrophotometer (Model lambda 25- Perkin Elmer Company), Eppendorf versatile 5810 series centrifuge and pH meter (Sense Ion 378, Hack) were used during process.
Data format	Analyzed
Experimental factors	The contact time, initial COD concentration and solution pH were studied for the removal of MTBE In the SBR reactor.
Experimental features	The main substrate of the synthetic solutions for to feed the SBR reactor, including methanol, TBA and MTBE, along with nutrients (nitrogen and phosphorus), and essential elements (micro elements) was injected to during application of reactor.
Data source location	Ahvaz city, Khuzestan province, Iran
Data accessibility	available in this article

**Value of the data**

• Data showed that mixed microbial mass is able to degrade high concentration of methanol and MTBE in petroleum wastewater.
• According to data SBR can be used as a biological treatment method to remove MTBE in petroleum wastewater.
• Removal of MTBE using SBR has no need for extra tanks, secondary sedimentation and system of returning sludge.

## 1 Data

**Fig. 1:**
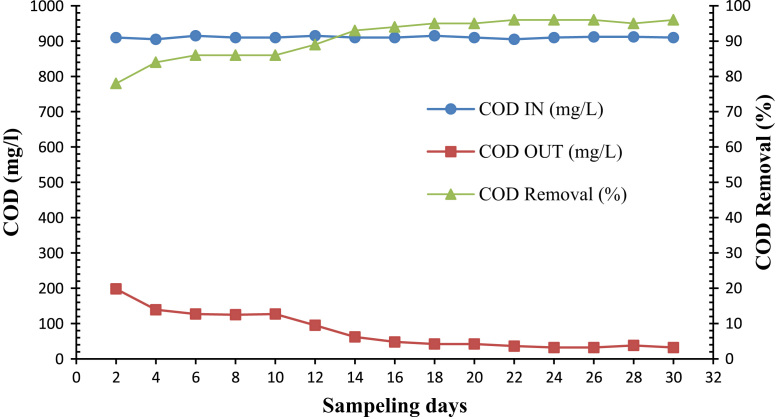
Trend of COD removal in the SBR during feeding the reactor by methanol (32 d).

**Fig. 2:**
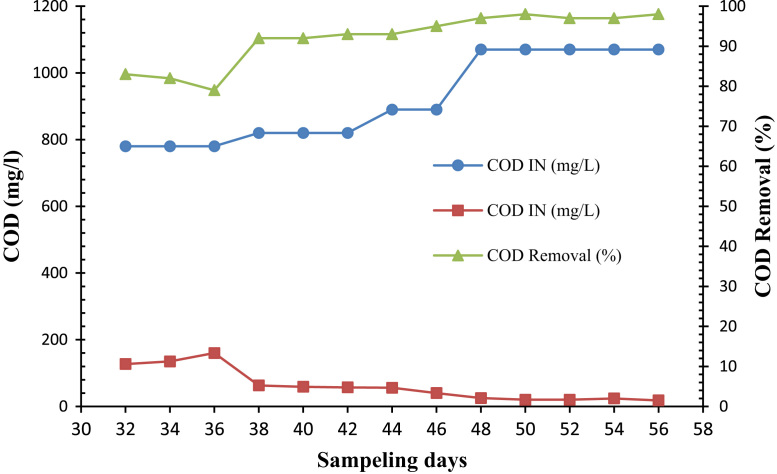
Trend of COD removal in the SBR during feeding the reactor by TBA (24 d).

**Fig. 3:**
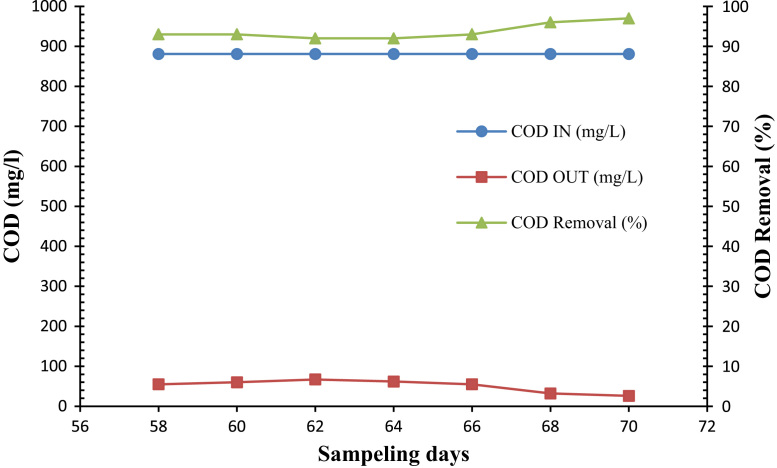
Trend of COD removal in the SBR during the reactor feeding by MTBE (12 d).

**Fig. 4:**
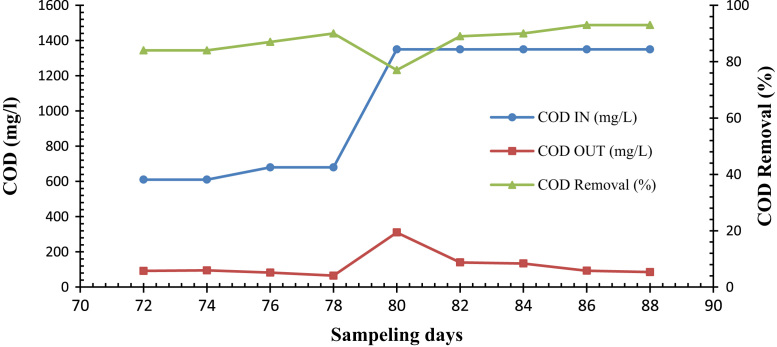
Trend of COD removal in the SBR during feeding the reactor by real wastewater of petroleum (16 d).

Microorganisms for biodegradation of MTBE were isolated from the real petrochemical wastewater treatment plant. Nutrient injection values were calculated regard to input COD concentration. As shown in Table 1 an environment with natural pH is a promising condition for the maximum growth rate of bacteria. Fig. 1, Fig. 2, Fig. 3, Fig. 4 show the trend of COD removal by microorganisms in 4 steps. At first step methanol was used as substrate and then in second and third steps, TBA and MTBE was used as substrate, respectively. We also collected data when used reactor for COD removal of real petroleum wastewater. In this study the concentration of COD during the process varied in the range of 313–780 mg/l. At the all four steps, change in the pH of treated wastewater was very low.

**Table 1:** pH changes in the SBR for removal of organic matter in different feeding steps including methanol (30 d), MTBA (24 d), TBA (12 d) and real wastewater of petroleum (16 d).

Removal methanol		Removal MTBA		Removal TBA		Removal MTBA and by real wastewater of petroleum	
Time (day)	pH	Time (day)	pH	Time (day)	pH	Time (day)	pH
2	7.34	32	7.45	58	7.62	72	7.62
4	7.2	34	6.95	60	7.57	74	7.95
6	7.36	36	7.16	62	7.34	76	8.09
8	7.27	38	7.12	64	7.3	78	8.11
10	7.52	40	7.16	66	7.44	80	8.22
12	7.52	42	8.34	68	7.65	82	8.06
14	7.24	44	8.04	70	7.52	84	7.96
16	6.92	46	7.45			86	8.04
18	7.2	48	7.74			88	8.11
20	7.19	50	7.82				
22	8.55	52	7.74				
24	8.12	54	7.68				
26	8.14	56	7.6				
28	8.22						
30	8.08

## 2 Experimental design, materials, and methods

In this study, all chemicals materials were purchased from Merck. Microorganisms for biodegradation of MTBE were isolated from the real petrochemical wastewater treatment plant. The SBR reactor, a cylindrical shape with a thickness of 2 mm, height of 50 cm, diameter of 12 cm and volume of 6.1, was made of glass. It was complemented by two peristaltic pumps for feeding and discharging effluent and excess sludge [1], [2], [3]. H_2_SO_4_ and NaOH were used to adjust pH in neutral state of 7. Present work has shown that an environment with natural pH is a promising condition for the maximum growth rate of bacteria (Fig. 1) [4], [5]. 70 mg/l of MTBE was determined as the optimal loading for SBR in this study. The main substrate for feeding the SBR was included methanol, TBA and MTBE along with nutrients (nitrogen and phosphorus). Gas Chromatograph (Agilent model) was used for measuring the MTBE [6], [7], [8]. Low levels of essential elements (micro elements) were injected to SBR during application. In order to prepare activate and adapted microorganisms, the primary activated sludge (Seed sludge) was collected from the petrochemical Complex [9]. According to Table 2, Chemical oxygen demand (COD) was analyzed with standard methods (3249) for evaluation of efficiency of process [10].

**Table 2:** The process of analyzing of COD.

• Add adequate sample to COD balloon • Add HgSO_4_ • Add sulfuric acid • Swirl until all the mercuric sulfate has dissolved • Sulfuric acid-silver sulfate solution • gently swirl until the solution is thoroughly mixed • Glass beads should be added to the reflux mixture to prevent bumping • Attach the flask to the condenser and reflux the mixture for two hours	• Cool, and wash down the interior of the condenser with 25 mL of distilled water • Titrate with standard ferrous ammonium sulfate (FAS) • Run a blank, using 50 mL of distilled water in place of the sample together with all reagents and subsequent treatment.
	Calculation:
	$$\underset{\frac{mg}{L}}{COD}=\frac{(A-B)\times N\times 8000}{V}$$
	*A*=ml FAS consumption for blank
	*B*=ml FAS consumption for sample
	*N*=normality of FAS
	*V*=Volume of sample (ml)
