# Supplementary material for: Efficiency of sequencing batch reactor for removal of organic matter in the effluent of petroleum wastewater
Source: Data Brief. 2018 Jul 4;19:2041–6. doi: 10.1016/j.dib.2018.06.094 (PMC6141387; doi:10.1016/j.dib.2018.06.094)
Supplement: Supplementary file 1 — Supplementary material. [file mmc1.docx]

Dear editor-in-Chief

Your comments and those of the reviewers were highly insightful and enabled us to greatly improve the quality of our manuscript. In the responses to reviewer file, has been presented our point-by-point responses to each of the comments of the reviewers as well as your own comments. Revisions in the text are shown using colored font for correcting. Please find attached responses to reviewers' comments file and revised version of our manuscript entitled “**Efficiency of sequencing batch reactor for removal of organic matter in the effluent of petroleum wastewater**”. We would like to resubmit for publication as data article in **Data in brief**. We hereby that this manuscript has not submitted else journal.

All author declare that there are not any interest of conflict.

We hope that the revisions in the manuscript and our accompanying responses will be sufficient to make our manuscript suitable for publication in Journal of **Data in brief**.

We shall look forward to hearing from you at your earliest convenience.

Yours sincerely,

Dr. Mehdi Vosoughi
